# The Cellular and Molecular Determinants of Naphthoquinone-Dependent Activation of the Aryl Hydrocarbon Receptor

**DOI:** 10.3390/ijms21114111

**Published:** 2020-06-09

**Authors:** Samantha C. Faber, Sara Giani Tagliabue, Laura Bonati, Michael S. Denison

**Affiliations:** 1 Department of Environmental Toxicology, University of California, Davis, CA 95616, USA; scfaber@ucdavis.edu; 2 Department of Earth and Environmental Sciences, University of Milano-Bicocca, 20126 Milan, Italy; s.gianitagliabue@campus.unimib.it (S.G.T.); laura.bonati@unimib.it (L.B.)

**Keywords:** aryl hydrocarbon receptor, naphthoquinones, ligand binding, docking analysis

## Abstract

1,2-naphthoquinone (1,2-NQ) and 1,4-naphthoquinone (1,4-NQ) are clinically promising biologically active chemicals that have been shown to stimulate the aryl hydrocarbon receptor (AhR) signaling pathway, but whether they are direct or indirect ligands or activate the AhR in a ligand-independent manner is unknown. Given the structural diversity of AhR ligands, multiple mechanisms of AhR activation of gene expression, and species differences in AhR ligand binding and response, we examined the ability of 1,2-NQ and 1,4-NQ to bind to and activate the mouse and human AhRs using a series of in vitro AhR-specific bioassays and in silico modeling techniques. Both NQs induced AhR-dependent gene expression in mouse and human hepatoma cells, but were more potent and efficacious in human cells. 1,2-NQ and 1,4-NQ stimulated AhR transformation and DNA binding in vitro and was inhibited by AhR antagonists. Ligand binding analysis confirmed the ability of 1,2-NQ and 1,4-NQ to competitively bind to the AhR ligand binding cavity and the molecular determinants for interactions were predicted by molecular modeling methods. NQs were shown to bind distinctly differently from that of 2,3,7,8-tetrachlorodibenzo-*p*-dioxin (TCDD) and differences were also observed between species. Mutation of amino acid residues (F289, M334, and M342) involved in critical NQ:AhR binding interactions, decreased NQ- and AhR-dependent gene expression, consistent with a role for these residues in binding and activation of the AhR by NQs. These studies provide insights into the molecular mechanism of action of NQs and contribute to the development of emerging NQ-based therapeutics.

## 1. Introduction

**Figure 1:**
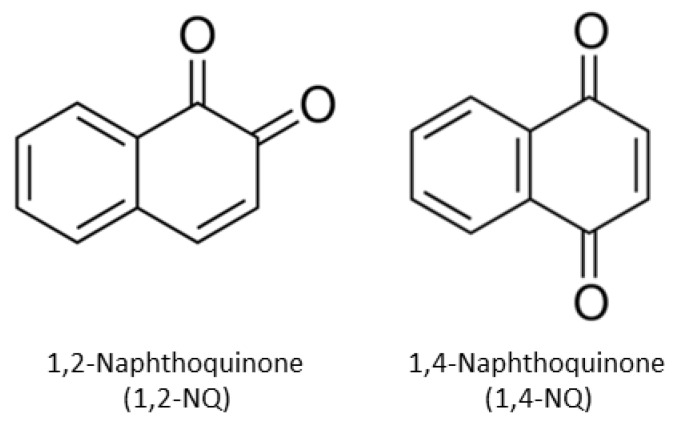
The chemical structures of 1,2-naphthoquinone (1,2-NQ) and 1,4-naphthoquinone (1,4-NQ).

Naphthoquinones (NQ) are byproducts of polycyclic aromatic hydrocarbon (PAH) oxidation and photolysis reactions and have been identified in a wide variety of materials (i.e., botanicals, air pollutants, and biologics) [1,2,3,4,5,6]. Mechanistically, NQ act as redox cyclers to stimulate the generation of free radicals involved in diverse physiological and pathophysiological outcomes [1,2,7], such as DNA damage and alterations of vital metabolic pathways (i.e., cellular respiration). The ability of NQs to act as potent cytotoxic, antiproliferative, and antimicrobial agents has led to an interest in designing NQ-based anticancer agents [1,2,3,8,9,10,11] and 1,2-naphthoquinone (1,2-NQ) and 1,4-naphthoquinone (1,4-NQ) are considered particularly promising in this regard. Although 1,2-NQ and 1,4-NQ are structurally similar (Figure 1), differences in the position of the carbonyl groups have been shown to result in diverse biological activity [1,9,10,12,13], and development of these compounds for practical applications require improved understanding of the molecular mechanisms underlying their modes of action.

The aryl hydrocarbon receptor (AhR) is a ligand-dependent transcription factor that regulates the expression of a wide variety of genes and it plays a pivotal role in a broad spectrum of biological, physiological and toxicological effects [14,15,16,17,18,19]. While the best studied high affinity ligands (agonists) for the AhR include polycyclic and halogenated aromatic hydrocarbons (PAHs and HAHs, respectively), the AhR can bind and be activated by structurally diverse ligands and cross species differences in AhR ligand specificity have been observed [15,20,21,22,23,24]. Localized in the cytosol as part of a multiprotein complex, agonist binding to the AhR stimulates translocation of the AhR protein complex into the nucleus, where dimerization of the AhR with its binding partner, the aryl hydrocarbon nuclear translocator (ARNT), releases the ligand-bound AhR from its associated proteins and converts the AhR into its DNA binding form [15,24,25]. Binding of the ligand: AhR:ARNT complex to its specific DNA binding site, the dioxin response element (DRE), results in transcriptional activation of adjacent genes [15,21,24,25,26]. Notably, while naphthalene (NA) (the precursor to both 1,2-NQ and 1,4-NQ) reportedly fails to bind to and/or activate the AhR, 1,4-dihydroxy-2-naphthoic acid and several related NA derivatives were shown to be able to act as AhR agonists/antagonists [27]. Additionally, numerous polychlorinated NAs (PCNs) have been shown to be AhR agonists and some higher chlorinated congeners (those with five or more chlorines) have physicochemical characteristics similar to the high affinity and prototypical HAH AhR ligand, 2,3,7,8-tetrachlorodibenzo-*p*-dioxin (TCDD, dioxin), and can stimulate both AhR-dependent gene expression and produce TCDD-like toxic effects [28].

Highly reactive quinone metabolites are known to stimulate electrophilic signal transduction pathways by their ability to covalently modify and activate transcription factors such as nuclear factor erythroid 2-related factor 2 (NRF2) [1,2]. Recently, quinone metabolites of NA (1,2-NQ and 1,4-NQ) as well as 1,4-benzoquinone, tert-butyl-1,4-benzoquinone, and other electrophilic mono- and di-cyclic quinones with covalent binding capacity were observed to upregulate CYP1A1/cyp1a1 mRNA in human HepG2 and mouse hepa1c1c7 cells, while the parent compounds had little effect [29,30]. Further, some quinones, including 1,2-NQ and 1,4-NQ, simulate AhR nuclear translocation and induction of cyp1a1 in mouse hepatoma cells in an AhR-dependent manner and the induction could be blocked by an AhR antagonist. Interestingly, 1,2-NQ was observed to covalently bind to the AhR, stimulate AhR binding to the cyp1a1 promoter region and induce DRE-dependent reporter gene induction. This led to the hypothesis that 1,2-NQ and other quinones activated the AhR via covalent modification of a thiol group in the AhR, presumably with one residue (C327) contained within the ligand binding pocket [29]. Nevertheless, the distinct mechanism(s) of AhR activation and whether these compounds bind within the AhR ligand binding domain (LBD) is unknown. Considering the significant differences in physicochemical characteristics of 1,2-NQ and 1,4-NQ from known high affinity AhR agonists, coupled with the lack of results demonstrating direct competitive binding of these chemicals to the AhR LBD, raises questions as to whether these quinones are direct or indirect ligands for the AhR or if they activate the AhR in a ligand-independent manner. In addition, given the role of 1,2-NQ and 1,4-NQ as active functional groups in promising therapeutics, investigation of the cross-species similarities and differences in AhR binding is also warranted.

Recent evidence has shown that the addition of electrophilic (reactive oxygen species (ROS)-generating) chemicals such as methylated pentavalent arsenic metabolites [31] or a quinone derivative of a non-dioxin-like polychlorinated biphenyl (4-chlorobiphenyl [32]) to cells in culture can stimulate AhR nuclear translocation and induction of cyp1a1 and DRE-dependent reporter gene expression, and these responses can be inhibited by an AhR antagonist. However, since none of the tested arsenicals or 4-chlorobiphenyl metabolites were shown to be competitive AhR ligands, these compounds appeared to activate the AhR and AhR signaling pathway in a ligand-independent manner. Ligand-independent activation of the AhR and AhR-dependent induction of cyp1a1 by a variety of chemicals and signal transduction pathways has been previously reported [33,34,35]. Additionally, since the oxidant H_2_O_2_ has been shown to stimulate the formation of tryptophan oxidation products (6-formylindolo[3,2-b]carbazole (FICZ) and others) that are extremely high affinity AhR agonists [36,37], indirect activation of the AhR by chemicals that can generate reactive oxygen species could explain or partially explain AhR activation by quinones as well as the methylated arsenical metabolites. Additionally, chemical-dependent inhibition of CYP1A1/cyp1a1 activity reportedly can lead to activation of the AhR and AhR-dependent gene expression by the low levels of metabolically labile, but highly potent AhR agonists (i.e., FICZ) that can be found in cell culture media [38]. Whether this CYP1A1/cyp1a1 inhibitory mechanism plays any role in the induction of AhR signaling by 1,2-NQ, 1,4-NQ or other reactive quinones is currently unknown. Here we have confirmed that 1,2-NQ and 1,4-NQ can stimulate AhR-dependent gene expression in a species-specific manner and have extended these analyses to examine their ability to directly bind to and activate the AhR in vitro, using a combination of ligand and DNA binding assays and in silico ligand-docking analysis.

## 2. Results

### 2.1. 1,2-NQ and 1,4-NQ Induce AhR-Dependent Gene Expression in Mouse and Human Cells

**Figure 2:**
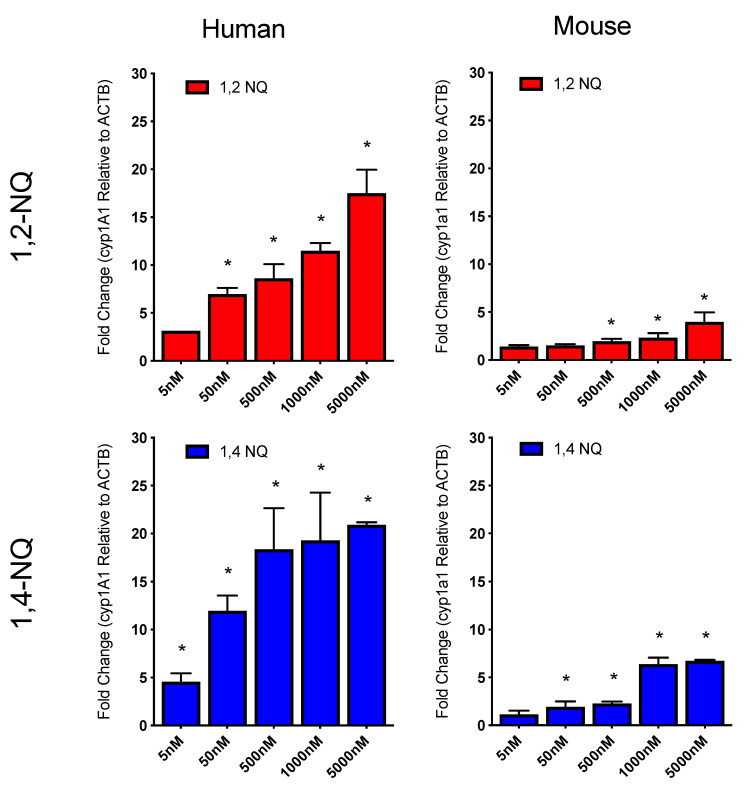
1,2-NQ and 1,4-NQ induce CYP1A1/cyp1a1 mRNA in mouse and human hepatoma cells. Human HG2L7.5c.1 and mouse H1L7.5c.3 hepatoma cells were incubated with DMSO (1% *v*/*v*), 1,2-NQ (5 nM–5 μM) or 1,4-NQ (5 nM–5 μM). After 4h incubation, cells were harvested with TRIzol reagent, RNA isolated, cDNA reverse transcribed and CYP1A1/1a1 mRNA transcripts quantitated by qPCR as described under Materials and methods. CYP1A1 and cyp1a1 transcript was normalized to beta-actin reference gene control and background DMSO was subtracted. Values were expressed as the mean ± standard deviation of triplicates of three biological replicates cells and an asterisk (*) indicates those values that were significantly different from DMSO treated cells at *p* < 0.05 as determined by Two-Way ANOVA.

1,2-NQ and 1,4-NQ were previously reported by Akibo et al. [29,30] to activate the AhR and induce AhR-dependent gene transcription in mice. Prior to more extensive analysis of the specific interactions of these compounds with the AhR, we first confirmed that 1,2-NQ and 1,4-NQ stimulated induction of the AhR-responsive gene CYP1A1/cyp1a1 in human and mouse hepatoma cells, respectively, in a concentration-dependent manner (Figure 2). In human (HG2L7.5c1) cells, 1,2-NQ and 1,4-NQ exposure induced CYP1A1 mRNA levels by ~20-fold, whereas these compounds only induced cyp1a1 mRNA levels by ~5-fold in mouse (H1L7.5c3) cells. These results not only showed that 1,2-NQ and 1,4-NQ were more potent inducers of CYP1A1 in human cells relative to cyp1a1 in mouse cells, but that 1,4-NQ was somewhat more potent than 1,2-NQ. Thus, these studies confirm the ability of 1,2-NQ and 1,4-NQ to simulate induction of the AhR-responsive CYP1A1/cyp1a1 gene in human and mouse cells.

### 2.2. NQ-Mediated Induction of AhR-Dependent Gene Expression Requires ARNT

**Figure 3:**
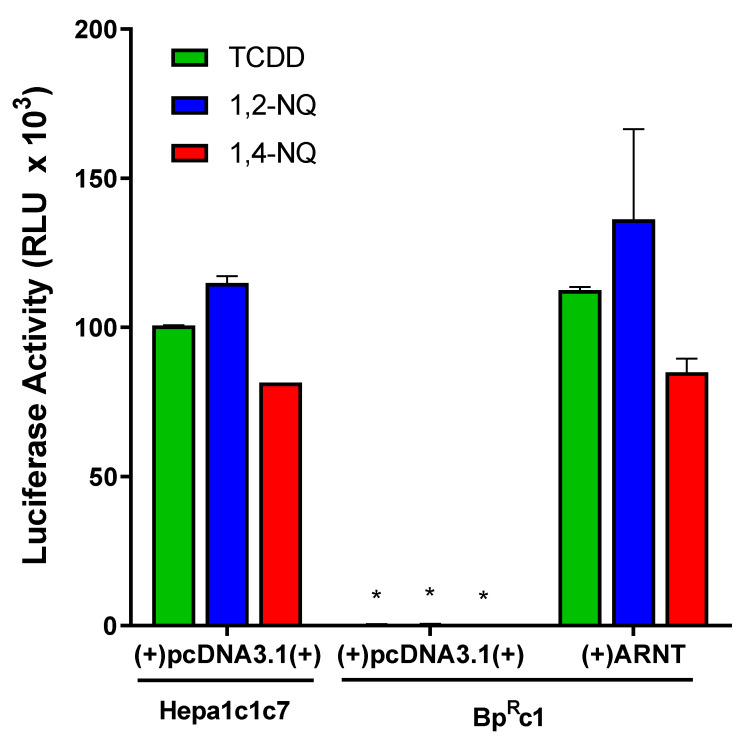
Aryl hydrocarbon nuclear translocator (ARNT) is required for aryl hydrocarbon receptor (AhR)-dependent gene induction by 1,2-NQ and 1,4-NQ. Wild-type and ARNT-defective (Bp^R^c1) mouse hepatoma (Hepa1c1c7) cells were transiently transfected with control pcDNA3.1(+) or ARNT expression vector and the AhR-responsive luciferase reporter plasmid pGudLuc6.1. After 24h, cells were incubated with DMSO (0.1%, *v*/*v*), 2,3,7,8-tetrachlorodibenzo-*p*-dioxin (TCDD) (10 nM), 1,2-NQ (5 μM), or 1,4-NQ (5 μM) for 18 to 22 h. Luciferase activity was measured and corrected for background luciferase activity (DMSO). Values were expressed as the mean ± standard deviation of triplicates of three biological replicates and an asterisk (*) indicates those values that were significantly different from wild-type Hepa1c1c7 cells at *p* < 0.05 as determined by Two-Way ANOVA.

The canonical AhR gene induction pathway requires AhR heterodimerization with ARNT for DNA binding and downstream gene transcription, although alternate AhR-dependent and ARNT-independent mechanisms of gene expression have been reported [39]. While previous cell-free experiments reported the ability of 1,2-NQ to stimulate binding of his-tagged ARNT with luciferase-tagged AhR [29,30], the direct involvement of ARNT in NQ-mediated gene induction in intact cells was not examined. To demonstrate a requirement for ARNT in 1,2-NQ- and 1,4-NQ-dependent induction of AhR-responsive gene expression, we examined the ability of 1,2-NQ and 1,4-NQ to induce AhR-dependent reporter gene expression in wild-type (Hepa1c1c7) and ARNT-deficient (BP^R^c1) mouse hepatoma cells [40]. Cells were transiently transfected with the AhR-responsive luciferase reporter construct pGudLuc6.1 and either an empty vector (pcDNA3.1) or an ARNT expression vector and TCDD-, 1,2-NQ, or 1,4-NQ induction of luciferase reporter gene activity determined. As expected, while 1,2-NQ, 1,4-NQ, and TCDD induced luciferase activity in wild-type mouse cells that contain endogenous levels of ARNT, no induction was observed in the ARNT-deficient mouse cells (Figure 3). However, contransfection of ARNT-deficient cells with an ARNT expression vector, restored the ability of TCDD, 1,2-NQ, and 1,4-NQ to stimulate luciferase reporter gene activity to levels comparable to that of wild-type mouse hepatoma cells. Together, these results confirm that ARNT is required for the ability of 1,2-NQ and 1,4-NQ to stimulate AhR-dependent gene expression and are consistent with NQs activating AhR-dependent gene expression through the canonical AhR:ARNT:DRE signaling mechanism.

### 2.3. 1,2-NQ and 1,4-NQ Directly Stimulate AhR Transformation and DNA Binding

**Figure 4:**
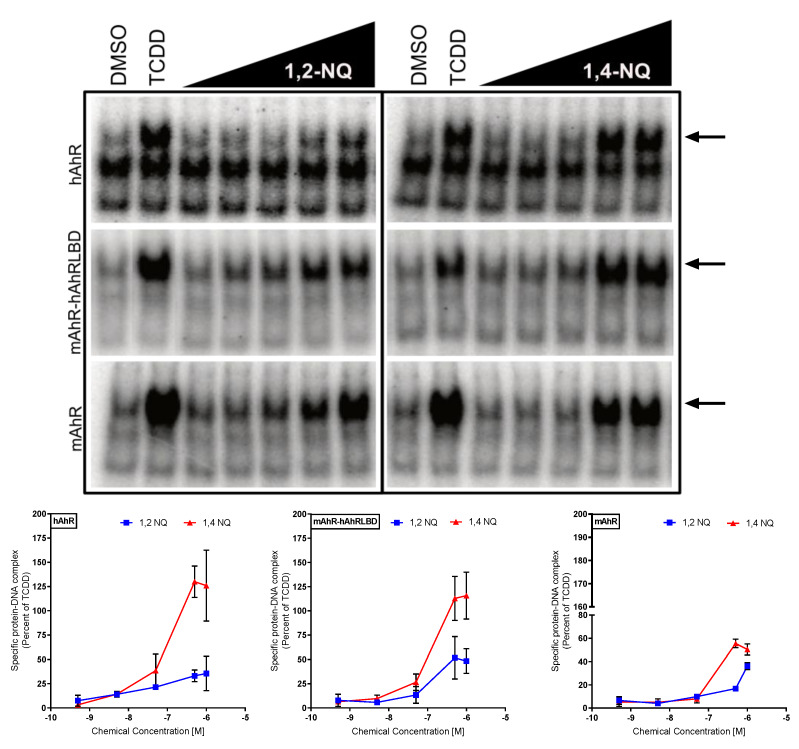
1,2-NQ and 1,4-NQ directly stimulate mouse and human AhR transformation and DNA binding in vitro. In vitro synthesized hAhR, mAhR-hAhRLBD, or mAhR and hARNT or mARNT were incubated in the presence of DMSO (1%, *v*/*v*), TCDD (20 nM), 1,2-NQ (0.5 nM–1 μM), or 1,4-NQ (0.5 nM–1 μM) for 2 h and protein-DNA complexes were detected by gel retardation analysis. Inducible AhR:ARNT:[^32^P]dioxin response element (DRE) complexes (indicated by the arrows) were quantified using Fujifilm MultiGauge software and values corrected for the amount of protein: DNA complex in the presence of DMSO (control), and then normalized to the amount of complex induced by TCDD. Values represent the mean ± standard deviation of three exposure replicates and three experiments and representative gels are shown.

To assess direct activation of the AhR by 1,2-NQ and 1,4-NQ, we examined their ability to stimulate AhR transformation and DNA binding. In vitro synthesized mouse AhR (mAhR), human AhR (hAhR), or the chimeric mAhR-hAhRLBD (a mAhR that contains the hAhR ligand binding domain [23]) and human or mouse ARNT (hARNT or mARNT, respectively) were incubated with DMSO, TCDD, and increasing concentrations of 1,2-NQ or 1,4-NQ. The mAhR-hAhRLBD chimera was used in these experiments to determine whether the LBD was responsible for, or contributes to, the enhanced activity of the NQs of the hAhR. In addition, these results will be able to be directly compared to the subsequent [^3^H]TCDD competitive ligand binding analysis experiments which must carried out using the mAhR-hAhRLBD chimera instead of the wild-type hAhR because of the extremely lability of the hAhR which makes ligand binding analysis of wild-type hAhR problematic [41]. AhR:ARNT:[^32^P]DRE complex formation determination (gel retardation analysis) revealed that 1,2-NQ and 1,4-NQ stimulated transformation and DNA binding of the human, mouse and mAhR-hAhRLBD in a concentration-dependent manner (Figure 4). In all cases, 1,4-NQ was a more potent and efficacious activator of AhR transformation and DNA binding than 1,2-NQ. The enhanced efficacy of NQ-induced transformation and DNA binding observed with the mAhR-hAhRLBD, compared to the mAhR, indicates that the hAhR LBD is responsible, at least in part, for the enhanced DNA binding response observed with NQ activated hAhR. Additionally, co-incubation with the species- and ligand-selective AhR antagonists CH223191, Stem Reginin 1, or 6,2′,4′,-trimethoxyflavone [14,42,43] resulted in a significant reduction in 1,2-NQ- and 1,4-NQ-dependent AhR transformation and DNA binding of all three AhRs (Supplemental Figure S1). Together, these in vitro transformation and DNA binding results not only are consistent with 1,2-NQ and 1,4-NQ being direct activators/ligands of the AhR ligands, but they also suggest that they interact with residues within the AhR ligand binding cavity differently than that of the high affinity AhR agonist, TCDD.

### 2.4. 1,2-NQ and 1,4-NQ Compete with [3H]TCDD for Binding to the Mouse and Human AhR

**Figure 5:**
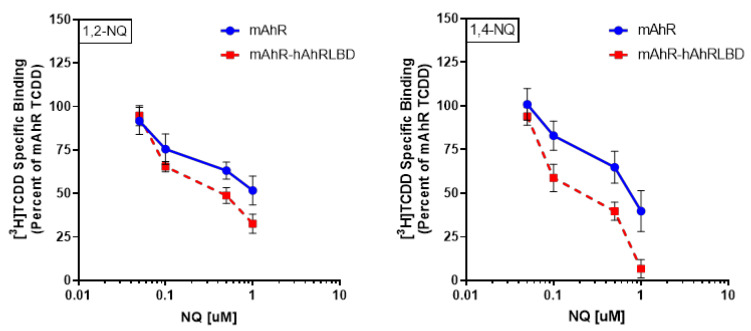
1,2-NQ and 1,4-NQ competitively bind to the human and mouse AhR ligand binding domain. In vitro synthesized mAhR or mAhR-hAhRLBD was incubated with 20 nM [^3^H]TCDD in the absence or presence of 1,2-NQ (50 nM–1 μM) (red) or 1,4-NQ (50 nM–1 μM) (blue) for 30 min, and [^3^H]TCDD bound to the protein fraction was measured by the hydroxyapatite binding assay. Unprogrammed lysate was used as a nonspecific binding control, and specific binding was calculated as the difference between the amount of [^3^H]TCDD bound in the total and nonspecific reactions. Values were normalized to the total specific binding of [^3^H]TCDD in the absence of competitor (set at 100%) and values represent the mean ± standard deviation of triplicate analyses of three incubations and are representative of three individual experiments.

To demonstrate that 1,2-NQ and 1,4-NQ act as AhR ligands by binding to the AhR ligand binding pocket, we examined their ability to directly compete with [^3^H]TCDD specific binding to the mAhR and mAhR-hAhRLBD. The human AhR is extremely labile and degrades quickly, making ligand binding analysis of wild-type hAhR difficult [41]. The enhanced stability of the hAhR LBD relative to wild-type hAhR in the mAhR-hAhRLBD chimera allows quantification of [^3^H]TCDD specific binding to the hAhR LBD [23]. The results in Figure 4 demonstrate that the human LBD present in the mAhR-hAhRLBD chimera was responsible for enhanced activity of NQs similar to that of wild-type hAhR and thus the chimera is an appropriate surrogate AhR for hAhR ligand binding analysis of NQs. These analyses demonstrated that both 1,2-NQ and 1,4-NQ competitively inhibit [^3^H]TCDD specific binding to the mAhR and mAhR-hAhRLBD in a concentration-dependent manner (Figure 5). Both NQs were also somewhat more effective competitors of [^3^H]TCDD binding to the mAhR-hAhRLBD compared to the mAhR, indicating that these NQs have a higher relative affinity for the hAhR LBD. Overall, these results confirm that 1,2-NQ and 1,4-NQ are direct competitive AhR ligands for the mouse and human AhR LBDs and these results are consistent with the observed increased relative response of NQs on hAhR-dependent gene expression and DNA binding (Figure 3 and Figure 4).

### 2.5. 1,2-NQ and 1,4-NQ Bind within the mAhR and hAhR Ligand Binding Cavity Differently Than TCDD

The above results demonstrate that 1,2-NQ and 1,4-NQ are structurally unique AhR ligands that can directly bind to and activate the mouse and human AhR and AhR-dependent gene expression through the canonical AhR signaling pathway. However, differences in the ability of the three ligand- and species-specific AhR antagonists to inhibit the agonist activity of these NQs (Supplemental Figure S1), suggest that they interact with residues within the AhR ligand binding cavity differently from TCDD or other known AhR agonists. At first, we examined the hypothesis proposed by Abiko and coworkers [29] about formation of a covalent bond between 1,2-NQ and the thiol group of C327, by predicting the pKa of this residue in the 3D model of the mAhR LBD. The obtained pKa value of about 8.9 suggested that both the orientation of this side-chain into the buried binding cavity and the lack of proximal basic amino acids do not facilitate the thiol deprotonation at pH = 7. On this basis, we hypothesized non-covalent interactions between the residues lining the AhR internal cavity and the NQ ligands drive ligand binding and AhR activation, similar to that of TCDD and other aromatic compounds.

**Figure 6:**
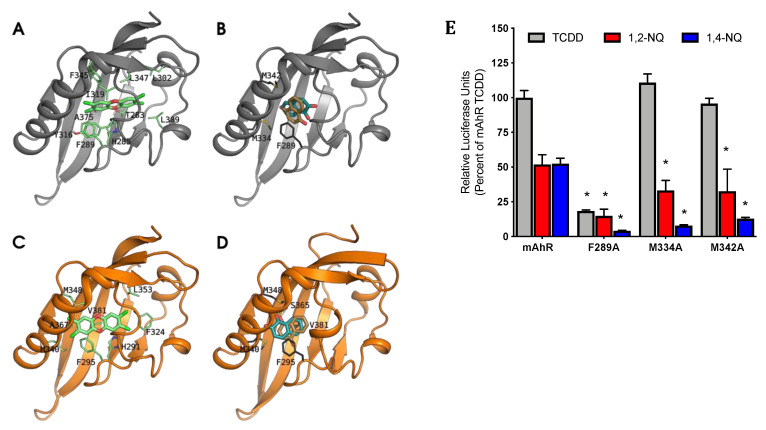
NQ interact with specific residues within the AhR binding cavity that are required for AhR functional activity. The models are shown as cartoons (mAhR in gray, hAhR in orange), and TCDD and NQs as sticks. The residues that mainly contribute to ΔG_bind_ are also shown as sticks (green for TCDD and dark gray for NQs). (**A**) TCDD pose in the mAhR LBD, taken as reference from previous analyses [44,45,46]: (**B**) NQs bind differently in the mAhR LBD compared to TCDD: the main interactions are with M334, M342, and F289. (**C**) TCDD pose in the hAhR: due to the steric hindrance of V381 both the position and the laying plane of the molecule within the cavity are different compared to the pose in mAhR. (**D**) The NQs poses in the hAhR are in the same relative position in the binding site as in the mAhR LBD, but additional stabilization provided by ligand interactions with V381 and S365 result in differences in NQ orientations. (**E**) COS-1 cells transiently transfected with wild-type or mutant mAhR pGudLuc6.1 were incubated with DMSO (0.1%, *v*/*v*), TCDD (10 nM), 1,2-NQ (5 μM), or 1,4-NQ (5 μM) for 18 to 22 h. Luciferase activity was measured, corrected for background activity (DMSO), and normalized to TCDD activity induced in mAhR transfected cells. Values represent the mean ± standard deviation of three independent experiments. Asterisks (*) indicate those values that are significantly reduced relative to that of mAhR at *p* < 0.05 as determined by Two-Way ANOVA.

Therefore, to verify our hypothesis of differential binding, we predicted the binding geometries of NQs by molecular docking to the mAhR LBD generated by homology modeling [44]. The results revealed that both 1,2-NQ and 1,4-NQ fit within the mAhR ligand binding cavity but with distinctly different orientations and residue interactions compared to that predicted for the binding of TCDD (Figure 6A,B). TCDD is predicted to occupy the central part of the mAhR cavity and interact with the most internal hydrophobic region of the ligand binding cavity via the 2-, 3-, or 7-, and 8-position chlorine atoms on TCDD, which reach the L302 and L309 residues [44]. In stark contrast, both NQs are predicted to bind at the entrance of the cavity and primarily interact with F289, M334 and M342 residues for stabilization (Figure 6B). While F289 is known to play a role in stability of TCDD within the mAhR cavity [45], the two identified methionine residues (M334 and M342) lie far from the TCDD-binding region, suggesting that NQs and TCDD have distinctly different mAhR binding features. Similar to the mAhR, NQ molecular docking within hAhR homology models [44] predicted that F295, M340, and M348 (F289, M334, and M342 in the mAhR) also contribute to NQ stabilization within the binding pocket (Figure 6D). The primary stabilization methionine residues for the NQs (M340 and M348) were predicted to interact also with TCDD within the hAhR cavity (Figure 6C,D). The results in Figure 5 indicate that NQs have a somewhat higher relative affinity for the hAhR compared to the mAhR, which is also reflective of the NQ-dependent AhR activation and gene expression results. Additional stabilization and enhanced affinity of NQs within the hAhR LBD was predicted to be due to hydrophobic interactions with V381 (A375 in the mAhR) and the hydrogen-bonding of a carbonyl oxygen atom with S365 (S359 in the mAhR) (Figure 6D). Molecular docking along with functional ligand binding, DNA binding, and gene expression analysis confirm that 1,2-NQ and 1,4-NQ bind and activate both the mAhR and hAhR and induce mAhR- and hAhR-dependent gene expression following interactions with key stabilizing residues within the AhR LBD. Stabilization by F295, M340, and M348, along with additional interactions with V381 and S365 are likely responsible for apparent increased affinity of NQs for the hAhR.

### 2.6. Mutation of F289, M334, and M342 Attenuates NQ-Mediated Induction of AhR-Dependent Gene Expression

To examine the functional role of the identified NQ ligand binding stabilizing residues in the mAhR LBD, F289, M334, and M342, each residue was mutated to alanine (A) and the impact of the mutations on NQ-stimulated AhR-dependent gene expression assessed. Mutation of these residues to alanine had no effect on expression levels relative to wild-type mAhR as previously determined via [^35^S]methionine-labeling and western blot analysis [22,46]. COS-1 cells were transiently transfected with pGudLuc6.1 and wild-type mAhR or mutant mAhR (with F289A, M334A, or M342A substitution) and incubated with TCDD, 1,2-NQ, or 1,4-NQ. As expected, cells transfected with wild-type mAhR demonstrated induction of AhR-dependent luciferase gene expression following incubation with TCDD, 1,2-NQ, or 1,4-NQ. In contrast, transfection with F289A mutant mAhR resulted in a ~75% decrease in TCDD-stimulated AhR-dependent luciferase gene activity and a decrease in 1,2-NQ and 1,4-NQ-stimulated reporter gene activity from 50% of TCDD to less than 15% (Figure 6E). While mutation of M334 and M342 to alanine had no significant effect on TCDD-stimulated AhR-dependent luciferase gene expression, both mutations decreased induction by 1,2-NQ by ~20% and that of 1,4-NQ by ~30–40% compared to wild-type mAhR. 1,4-NQ-stimulated AhR-dependent luciferase gene expression was more affected by the F289A, M334A, and M342A mutations compared to 1,2-NQ, suggesting differences in specific NQ binding interactions within the cavity. These results support the AhR homology model and molecular docking predictions for 1,2-NQ and 1,4-NQ and confirm that mutation of key stabilizing residues (F289, M334, and M342) within the mAhR alter NQ- and AhR-dependent gene expression.

## 3. Discussion

The emerging role of naphthoquinones in human disease and potential therapeutic applications have led to renewed interest in understanding the molecular mechanisms underlying NQ-dependent physiological and pathophysiological functions. In addition to its role in enhancing xenobiotic metabolism, the AhR mediates a wide variety of biological and toxicological pathways [14,15,16,17,18,19] and the molecular mechanism(s) of NQ-dependent AhR activation (i.e., direct, indirect, and/or ligand-independent) will aid in the development of novel therapeutics [11,31,32,33,34,35,37,38].

Utilizing a combination of gene expression, reporter gene analysis, gel retardation and ligand binding analysis, we demonstrate that 1,2-NQ and 1,4-NQ are full AhR ligands/agonists that can compete with [^3^H]TCDD for specific binding to the AhR, stimulate AhR transformation and DNA (DRE) binding in vitro and induce AhR-dependent induction of CYP1A1/cyp1a1 and DRE-driven reporter gene activity in human and mouse cell lines. Gene expression analysis not only revealed that 1,2-NQ and 1,4-NQ were more potent and efficacious AhR agonists in human cells compared to mouse cells, but that 1,4-NQ was slightly more potent than 1,2-NQ, consistent with its relative binding to the AhR. In addition, NQ-stimulated AhR transformation and DNA binding was greater with the hAhR compared to the mAhR and 1,4-NQ was a more efficacious activator of AhR transformation and DNA binding than 1,2-NQ in both species. Interestingly, the NQ-stimulated DNA binding results using the mAhR-hAhRLBD chimera, which contains the hAhR LBD in place of the mAhR LBD, were more comparable to DNA binding results with the hAhR than that with the mAhR, suggesting that the hAhR LBD is responsible for increased affinity, potency, and efficacy of AhR transformation and DNA binding observed in human cells. These species-specific differences in potency and efficacy of 1,2-NQ and 1,4-NQ likely result from their differential interactions with residues within the LBD of the AhR in each species.

The AhR is a promiscuous transcription factor that has been shown to bind and be activated by a wide range of structurally diverse ligands, and more recently, our group has described ligand-selective and species-specific AhR activation by the endogenous AhR ligand indirubin [23]. In the case of indirubin, differences in species-specificity and ligand-selectivity were shown to result from altered interactions between the ligand and residues within the human or mouse AhR ligand binding domain that resulted in altered AhR-dependent functional changes. This species-selectivity in ligand binding has also been observed with other structurally diverse AhR ligands [15,21,23]. To assess whether differences in binding interactions of NQs with residues within the mouse and human AhR LBDs account for their differential AhR response, we employed molecular docking simulations with established hAhR and mAhR homology models [44]. Simulation of the ligand binding event by molecular docking has previously allowed prediction of the TCDD binding geometry in both the mAhR and hAhR LBDs, and the main residues that contribute to the ligand:AhR complex stabilization were identified and confirmed by site-directed mutagenesis [44]. The same analysis was performed to predict the binding modes of 1,2-NQ and 1,4-NQ in both the mAhR and hAhR LBD in order to compare their arrangements within the binding cavity with that of TCDD, as well as to gain insights into possible species differences in NQ-dependent response. Despite similarities of NQs with TCDD and TCDD-like chemicals in some molecular features (e.g., coplanarity), NQs show smaller dimensions and higher polarity. Consequently, docking analysis indicated NQ binding geometries within the mAhR and hAhR binding cavity that were distinctly different from that of TCDD (Figure 5). TCDD binds deep within the cavity exhibiting stabilizing interactions both with residues in the middle of the cavity (i.e., the “TCDD-binding fingerprint”, that includes T283, H285, F289, Y316, I319, F345, and A375) [45] and with some hydrophobic residues (L302 and L309) lining the inner part of the cavity [44]. In contrast, both NQs bind at the entrance of the cavity, thanks to stabilizing interactions with M334 and M342, as well as some of the TCDD binding residues. Mutation of F289, M334, and M342 to alanine confirmed this prediction; only 1,2-NQ and 1,4-NQ binding was significantly decreased with the M334A and M342A mutants, while the F289A mutant AhR failed to respond to NQs or TCDD (Figure 6E). F289A has been previously shown to negatively affect ligand-dependent AhR transformation and DNA binding by structurally diverse ligands [22]. Other Authors recently published a docking study, including the treatment of protein flexibility, of a compound similar to 1,4-NQ, 1,4-dihydroxy-2-naphthoic acid (1,4-DHNA) [27], and predicted that this ligand binds the AhR in a more internal region of the binding cavity compared to 1,4-NQ. However, the difference in binding of 1,4-DHNA might be due to the additional salt-bridge and H-bonds formed by the 2-carboxyl group, which are absent with 1,4-NQ. Although docking analysis revealed some significant differences in the specific orientation of the NQ molecules at the entrance of the cavities of the mouse and human AhRs, it remains to be determined whether these differences in orientation contribute to the observed species differences in AhR response.

Previous TCDD docking simulations within the hAhR homology model have helped provide explanation as to the role of the internal hAhR residue, V381 (A375 in the mouse), in the reduced affinity of TCDD for the hAhR as compared to the mAhR. These studies indicated that the larger valine side-chain reduced the internal space required for accommodating the TCDD molecule [44]. Conversely, both 1,2-NQ and 1,4-NQ ligands were predicted to bind effectively into the modeled hAhR binding cavity, maintaining relative binding geometries similar to the mAhR (Figure 6D). Not only does V381 facilitate efficient binding by smaller ligands, such as the NQs, that occupy the entrance of the cavity, but the NQ:hAhR complexes also show enhanced stability compared to the mAhR, in agreement with the higher relative binding affinity for hAhR determined experimentally. Further, additional stabilization within the hAhR ligand binding cavity via hydrophobic interaction with V381 is consistent with quantitative structure-activity relationship (QSAR) studies identifying enhanced antiproliferative and cytotoxic capabilities of naphthoquinones with increased hydrophobicity [8,47].

Compared to 1,2-NQ, 1,4-NQ demonstrated a somewhat higher AhR binding affinity and a greater biological activity in terms of magnitude of AhR-dependent gene expression. Interestingly, examination of 1,2-NQ and 1,4-NQ as inhibitors of the ATPase domain demonstrated similar results with 1,4-NQ binding and acting as a more potent inhibitor of ATPase activity [8]. Further, simple structural changes in the location of functional groups along the benzene ring appear to alter the ability of quinone-based compounds to activate or suppress AhR activation. For example, Fukuda and coworkers [48] reported that the anthraquinone (AQ) compounds, juglone, 1,4-AQ, and anthralin, suppressed the TCDD-dependent rat cytosolic AhR transformation and DNA binding. Although AQs are structurally similar to NQs, AQs contain additional hydroxyl groups that are believed to confer suppressive AhR effects. In fact, levels of AQ inhibition are suggested to be enhanced by hydroxyl groups at the C1 or C4 position, which in conjunction with enhanced biological activity observed with 1,4-NQ, suggests that the C1 and C4 position increase stability through specific interactions with internal AhR residues for increased/decreased AhR transformation. Flavonoid compounds, which are structurally similar to NQ compounds but contain an additional heterocyclic ring at the C2 position (i.e., resveratrol, flavone, flavonol, and flavanone), also suppress DNA-binding activity of the AhR; however, flavonoids demonstrate an inverse correlation between number of hydroxyl groups and inhibitory effects [49]. Moreover, differential binding of NQ-based compounds can result in varied functional effects. Recently, the microbiota-derived hydroxylated quinoline derivative, 2,8-hydroxyquinoline (2,8-DHQ), was shown to modulate intestinal immune function through the canonical AhR signaling pathway and the hydroxyl groups were identified as critical structural determinants for AhR activation [50]. The diversity in AhR ligand binding has also been shown to result from species-specific differences in the amino acids within the LBD of the AhRs that may alter the structure of the binding cavity and the specific residues available for ligand binding [23,51,52]. Together, these studies may help explain why naphthalene, which does not contain additional functional groups (i.e., quinone or hydroxyl groups), fails to bind or activate the AhR, while naphthalene-like structures containing one or more functional groups (i.e., quinone, hydroxyl, or halogen groups) can interact with key amino acid residues to enable AhR binding and activation. The specific differences in AQ, NQ, and halogenated naphthalene compounds binding within the AhR is beyond the scope of this study, but future studies comparing the molecular dynamics of these compounds within the AhR binding cavity will aid in elucidating the mechanisms of binding of diverse naphthalene-based compounds within the AhR.

1,2-NQ and 1,4-NQ have been shown to influence oxidative status in rodent models and cells in culture, and have been linked to toxicity [1,2,6,53,54]. Their potential to activate the AhR signaling pathway in vivo could lead to increased production of reactive metabolites as a result of the induction of expression of AhR-responsive CYPs (e.g., CYP1A1, CYP1B1, CYP1A2, CYP2A5, etc.) as well as other enzymes known to be important in the metabolism and activation of naphthalene [2,55,56]; therefore, we investigated whether AhR antagonists can inhibit NQ-dependent activation of the AhR. Due to the extreme promiscuity of the AhR and diversity of ligands that can bind within the AhR ligand binding domain, known AhR antagonists have been shown to exhibit ligand-selective and species-specific inhibition of AhR function. CH223191 and TMF reportedly preferentially antagonize the ability of TCDD and TCDD-like HAHs, but not that of beta-naphthoflavone (BNF) and other flavonoid-like ligands, from binding to and activating the AhR and AhR-dependent gene expression [43]. In addition, the AhR antagonist SR1 is reported to be a human-selective AhR antagonist [14]. Interestingly, co-treatment of CH223191, SR1, or TMF resulted in repression of both 1,2-NQ- and 1,4-NQ-dependent AhR transformation and DNA binding of the mAhR, hAhR and the chimeric mAhR-hAhRLBD (Supplemental Figure S1). This suggests that each antagonist interacts with distinct residues within both the human and mouse AhR ligand binding cavity that effectively inhibits NQ-dependent AhR binding and/or transformation and is also consistent with the NQs binding differently within the LBD compared to that of TCDD.

Overall, the ability of 1,2-NQ and 1,4-NQ to directly bind to and activate the AhR and AhR signal transduction pathways have important implications for their biological, pharmacological, and toxicological effects. The findings from this study support preferential activation of the human AhR relative to the mouse, and the propensity of 1,2-NQ and 1,4-NQ to exhibit species-specific AhR activation. Future studies should investigate whether these species-specific effects can translate into diverse downstream AhR-dependent effects in order to aid in the development of novel risk mitigation strategies and therapeutic interventions.

## 4. Materials and Methods

### 4.1. Chemicals

TCDD was a gift from Dr. Stephen Safe (Texas A&M University). [^3^H]TCDD (13 Ci/mmol) was obtained from ChemSyn Laboratories (Lenexa, KS, USA) and [^32^P]-ATP (~6000 Ci/mmol) from Perkin-Elmer Life & Analytical Sciences (Waltham, MA, USA). Dimethylsulfoxide (DMSO) (≥ 99.9%), 1,2-NQ (97%) and 1,4-NQ (97%) were from Sigma-Aldrich Chemicals (St. Louis, MO, USA), CH223191 was from Chembridge Corporation (San Diego, CA, USA), StemRegenin (SR1) from Cayman Chemicals (Ann Arbor, MI, USA), and 6,2′,4′-trimethoxyflavone (TMF) was a gift from Dr. Gary Perdew (Pennsylvania State University, State College, PA, USA). All compounds were dissolved in DMSO.

### 4.2. Plasmids

mAhR and mouse ARNT (mARNT) expression plasmids, mβAhR/pcDNA3 and mβARNT/pcDNA3, respectively, have been previously described [22]. The chimeric mouse/human AhR expression plasmid, mAhR/hAhRLBD/pcDNA3, contains a full length mAhR cDNA in which the ligand binding domain (LBD) was replaced with the corresponding region of the hAhR LBD [23]. Human AhR and ARNT cDNAs were obtained from Christopher Bradfield (University of Wisconsin, Madison, WI, USA) and Oliver Hankinson (University of California, Los Angeles, LA, USA), respectively, and were inserted in pcDNA3β [57] to generate hβAhR/pcDNA3 and hβARNT/pcDNA3. Point mutations within the mβAhR/pcDNA3 mutant expression plasmids were generated with the QuikChange Lightning Site-Directed Mutagenesis Kit (Agilent Technologies, Santa Clara, CA, USA) and confirmed by sequencing.

### 4.3. AhR-Dependent Reporter Gene Induction in Transiently and Stably Transfected Cells

In transient transfection experiments, COS-1 (African green monkey kidney), Hepa1c1c7 (mouse hepatoma), and or BP^r^c1 (ARNT-deficient Hepa1c1c7 [40]) cells maintained in Minimal Essential Medium (MEM) (Sigma-Aldrich, St. Louis, MO, USA) with 10% fetal bovine serum (FBS) (Atlanta Biologicals, Flowery Branch, GA, USA) and 5% Penicillin/Streptomycin (P/S) (Gibco, Carlsbad, CA, USA) (10,000 U/mL) were plated in a 24-well culture plate at a density of 375,000 cells/well and allowed to attach overnight. Cells were transiently transfected (per well) with 20 ng wild-type mAhR (mAhR/pcDNA3.1(+)) or mutant AhR expression plasmids (in pcDNA3.1(+)), 20 ng of mARNT/pcDNA3.1(+) or pcDNA3.1(+) and 200 ng of the AhR-responsive luciferase reporter plasmid pGudLuc6.1 [58] using Lipofectamine 2000 (ThermoFisher Scientific, Waltham, MA, USA) [23]. Twenty-four hours after transfection, cells were incubated with DMSO (0.1%, *v*/*v*), or the indicated concentration of TCDD, 1,2-NQ, or 1,4-NQ for 18 to 22 h, followed by visual inspection of the cells for toxicity, washing of the cells with phosphate-buffered saline, addition of 50 μL of passive lysis buffer (Promega, Madison, WI, USA), and lysis of cells for 20 min at room temperature with shaking [59]. Luciferase activity in 50 μL aliquots was measured in an Orion microplate luminometer (Berthold Technologies, Baden-Württemberg, Germany) following automatic injection of stabilized luciferase reagent as described [59]. In stably transfected cell experiments, recombinant mouse (H1L7.5c3) and human (HG2L7.5c.1) hepatoma cells, which contain the stably transfected enhanced AhR-responsive reporter gene plasmid pGudLuc7.5 [60,61], were plated (75,000 cells/well) in 96-well plates and incubated at 37 °C for 24 h prior to chemical treatment. Cells were incubated with DMSO (1% (*v*/*v*)) or the indicated concentration of test chemical in DMSO for 4 h, followed by visual inspection for toxicity, lysis and measurement of luciferase activity in each well as previously described in detail [59]. Luciferase activity in treated cells was corrected for background luciferase activity present in DMSO-treated cells and values expressed as relative light units (RLUs) or as a percent of the luciferase activity obtained with the maximally inducing concentration of TCDD.

### 4.4. AhR DNA Binding (Gel Retardation) Assay

Wild-type and mutant AhRs and ARNT were synthesized in vitro in the presence of unlabeled L-methionine using rabbit reticulocyte lysate (Promega, Madison, WI, USA) as previously described [22,23]. The resulting AhR and ARNT translation mixtures and MEDGK buffer (25 mM MOPS (3-(N-morpholino)propanesulfonic acid; pH 7.5), 1 mM EDTA, 1 mM dithiothreitol, 10% (*v/v*) glycerol, 150 mM KCl) were mixed in a 1:1:8 (*v/v/v*) ratio and incubated with DMSO (1% final concentration) or the indicated concentration of TCDD, 1,2-NQ, or 1,4-NQ in DMSO, in the absence or presence of 10 μM of the desired AhR antagonist (CH223191, SR1, or TMF) (Sigma Aldrich, St. Louis, MO, USA) for the indicated period of time at room temperature. An aliquot of each incubation was mixed with [^32^P]-labeled double-stranded DRE3 containing oligonucleotide and protein-DNA complexes resolved by gel retardation analysis as described in detail [62]. Gels were visualized using a FLA-9000 Fujifilm Imager (Fujifilm Life Sciences, Cambridge, MA, USA) and protein-DNA complexes quantitated with Fujifilm MultiGauge software v3.0.

### 4.5. Hydroxyapatite [^3^H]TCDD Ligand Binding Assay

[^3^H]TCDD specific binding to wild-type mAhR and the chimeric mouse/human mAhR/hAhRLBD synthesized in vitro using rabbit reticulocyte lysate (Promega, Madison, WI, USA) was carried out as previously described [22). [^3^H]TCDD specific binding was determined by subtracting the amount of [^3^H]TCDD bound to unprogrammed lysate (nonspecific binding) from the total amount of [^3^H]TCDD binding to lysate containing in vitro expressed AhR. The amount of [^3^H]TCDD specific binding remaining in the presence of competitor chemical was expressed as a percent of the total [^3^H]TCDD specific binding.

### 4.6. Molecular Modeling

The mAhR and hAhR PASB LBD structures have been previously generated by homology modeling starting from the sequences: UniProt id P30561 and residues 278−384 for mAhR; UniProt id P35869 and residues 284−390 for hAhR. Three ligand-bound X-ray structures of the HIF-2α protein (PDB ID: 3F1O, 3H7W, and 3H82) were used as templates [44]. Four out of the one hundred models with the best DOPE score obtained by MODELLER (v. 9v7, University of California, San Francisco, CA, USA) [63] for each species were selected as representatives of the conformational variety of the modeled LBD by cluster analysis. Each model was refined by Molecular Mechanics (MM) energy minimization using MacroModel (Schrödinger Release 2015-4, Schrödinger, New York, NY, USA) [64] and maintaining a template ligand inside the binding cavity, as previously described [44]. The NQs molecular structures were downloaded from PubChem (1,4-NQ CID: 8530; 12-NQ CID: 10667), prepared with LigPrep (Schrödinger Release 2015-4, Schrödinger, New York, NY, USA) [65], and optimized by MM energy minimization. Molecular docking was carried out using Glide extra precision (XP) (Schrödinger Release 2015-4, Schrödinger, New York, NY, USA) [66,67]. An ensemble docking approach, based on ligand docking to the four representative conformations of the modeled receptor, was used to include receptor flexibility. To take into account the induced-fit effects of the NQs on the protein domain, a further optimization of the docking poses was performed using MacroModel [64]. Finally, the binding free energy (ΔG_bind_) of the obtained ligand-receptor complexes was calculated with the MM Generalized Born Surface Area (MM-GBSA) method [68] implemented in Prime MM-GBSA (Schrodinger Release 2015-4, Schrodinger, New York, NY, USA) [69] that includes evaluation of the solvation contributions by an implicit solvent model. Visualization of the models was accomplished using PyMOL (DeLano Scientific, San Francisco, CA, USA) [70].

### 4.7. Quantitative Real-Time Polymerase Chain Reaction

Recombinant mouse hepatoma H1L7.5c3 and HG2L7.5c.1 cells, maintained in MEM with 10% FBS and 5% P/S, were plated in 6-well plates (750,000 cells/mL) and allowed to attach for 24 h. DMSO (1% (*v*/*v*)) or the indicated concentration of TCDD, 1,2-NQ, or 1,4-NQ was added and the cells incubated for 4 h followed by isolation of total RNA using TRIzol reagent (ThermoFisher Scientific, Waltham, MA, USA). cDNA concentration was adjusted to 2 μg/μL and synthesis was performed using the High Capacity cDNA Reverse Transcription Kit (Applied Biosystems, Foster City, CA, USA) with a Bio-Rad T100 Thermal Cycler (thermal cycler conditions were 10 min 25 °C, 120 min 37 °C, 5 min 85 °C) (Bio-Rad, Hercules, CA, USA). qPCR was performed using the TaqMan Gene Expression Assay (Applied Biosystems, Foster City, CA, USA) with the TaqMan Fast Universal PCR Master Mix (ThermoFisher Scientific, Waltham, MA, USA), β-actin control (ID: Hs01054797_g1 (Human β-actin; catalog number 4331182) and Mm02619580_g1 (Mouse β-actin; catalog number 4331182)), and mouse Cyp1a1 (ID: Mm00487218_m1; catalog number 4331182) or human CYP1A1 (ID: Hs01054796_g1; catalog number 4331182) TaqMan Gene Expression Probes (Applied Biosystems, Foster City, CA, USA). The assays were performed in an Applied Biosystems 7500 Fast Sequence Detection System (ThermoFisher Scientific, Waltham, MA, USA), mRNA levels were normalized to β-actin and values expressed relative to β-actin mRNA levels in untreated cells (set to a value of 1.0) as per the delta-delta Ct (2^−ΔΔ*CT*^) method [59].

### 4.8. Statistical Analysis

Analysis of statistical significance of differences between experimental values was conducted using Two-Way ANOVA. EC_50_ and IC_50_ values were calculated using nonlinear regression (three-parameter) analysis in GraphPad Prism v. 8.1 (La Jolla, CA, USA).

## Supplementary Materials

Supplementary materials can be found at https://www.mdpi.com/1422-0067/21/11/4111/s1. Figure S1. NQ-stimulated AhR DNA binding is inhibited by AhR antagonists.

## Abbreviations

2:8-DHQ	2,8-hydroxyquinoline
1,2-NQ	1,2-naphthoquinone
1,4-NQ	1,4-naphthoquinone
AhR	aryl hydrocarbon receptor
AQ	anthraquinone
ARNT	aryl hydrocarbon nuclear translocator
BNF	beta-naphthoflavone
CYP	cytochrome P450
DMSO	dimethylsulfoxide
DRE	dioxin response element
FICZ	6-formylindolo[3,2-b]carbazole
HAH	halogenated aromatic hydrocarbons
hAhR	human AhR
LBD	ligand binding domain
MM	Molecular Mechanics
MM-GBSA	MM Generalized Born Surface Area
NQ	naphthoquinone
mAhR	mouse AhR
mAhR-hAhRLBD	mouse AhR containing the human AhR ligand binding domain
NRF2	nuclear factor erythroid 2-related factor 2
PAH	polycyclic aromatic hydrocarbons
PCN	polychlorinated naphthalene
QSAR	quantitative structure-activity relationship
TCDD	2,3,7,8-tetrachlorodibenzo-*p*-dioxin
TMF	6,2′,4′-trimethoxyflavone
