# Supplementary material for: The Cellular and Molecular Determinants of Naphthoquinone-Dependent Activation of the Aryl Hydrocarbon Receptor
Source: Int J Mol Sci. 2020 Jun 9;21(11):4111. doi: 10.3390/ijms21114111 (PMC7312509; doi:10.3390/ijms21114111)
Supplement: Supplementary file 1 [file ijms-21-04111-s001.pdf]

International Journal of
Molecular Sciences

Supplementary Materials

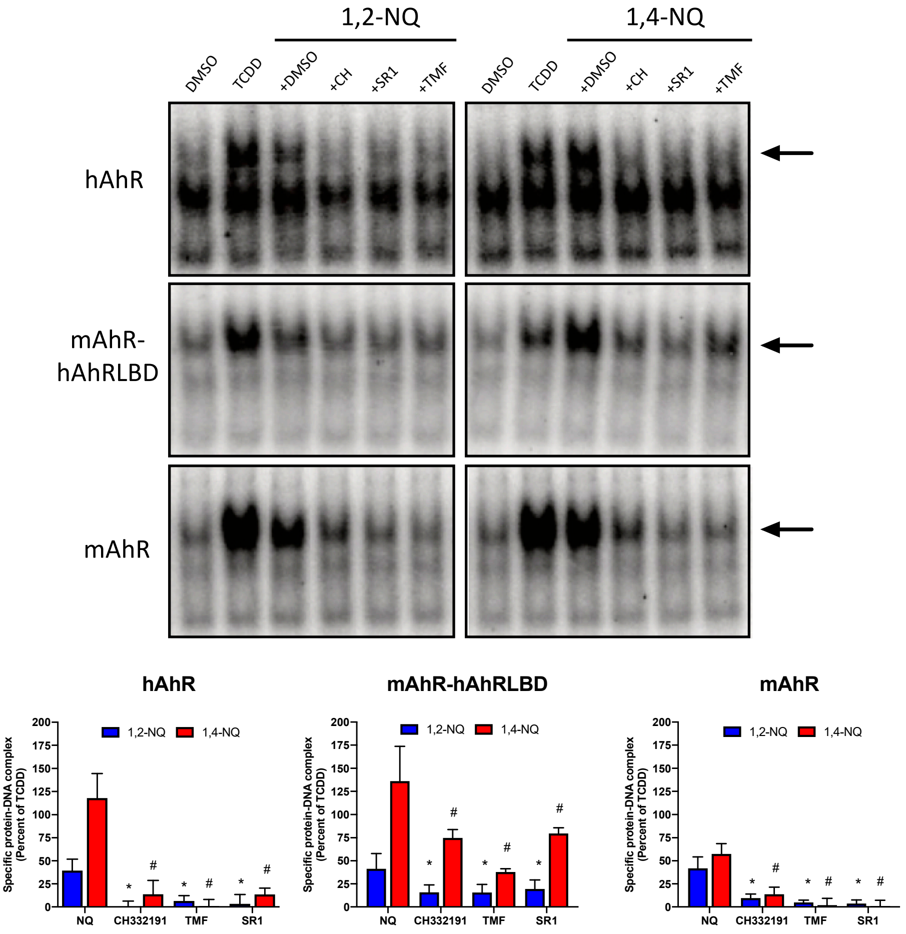

Figure S1. NQ-stimulated AhR DNA binding is inhibited by AhR antagonists. In vitro synthesized hAhR, chimeric mAhR-hAhRLBD, or mAhR and ARNT were incubated in the presence of DMSO (1%, *v*/*v*), TCDD (20 nM), 1,2-NQ (500 nM), or 1,4-NQ (500 nM) for 2 hours at room temperature in the absence or presence of AhR antagonists (CH223191 (10 μM), SR1 (10 μM), or TMF (10 μM)) and AhR:DNA complexes visualized by gel retardation analysis as described under Material and Methods. Representative gels are shown and the arrows indicate position of the induced AhR:ARNT:DRE complex. Inducible AhR:ARNT:[^{32}P]DRE complexes were quantified using Fujifilm MultiGauge software, values corrected for the amount of protein:DNA complex in the presence of DMSO (control), and then normalized to the amount of complex induced by TCDD. Values represent the mean ± standard deviation of three exposure replicates and three experiments. Asterisks (*) and number sign (#) indicate those values that are significantly reduced in the presence of an inhibitor relative to 1,2-NQ and 1,4-NQ, respectively.
